# Mycobiota of maize seeds revealed by rDNA‐ITS sequence analysis of samples with varying storage times

**DOI:** 10.1002/mbo3.609

**Published:** 2018-03-23

**Authors:** Hui‐Qin Xing, Jian‐Cang Ma, Bing‐Liang Xu, Shu‐Wu Zhang, Jin Wang, Li Cao, Xue‐Mei Yang

**Affiliations:** ^1^ College of Plant Protection Gansu Agricultural University and Biocontol Engineering Laboratory of Crop Diseases and Pests of Gansu Province Lanzhou Gansu China; ^2^ College of Agriculture and Biotechnology Hexi University Zhangye Gansu China; ^3^ Zhangye Maize Stock Production Base Zhangye Gansu China

**Keywords:** fungal diversity, ITS, maize, mycobiota, seeds

## Abstract

Fungi are an integral component of the plant microbiome. However, the composition and variation in the fungal communities (mycobiota) associated with seeds are poorly understood. In this study, we investigated the mycobiota of 11 maize seed samples with storage times ranging from 6 months to 12 years. Mycobiota were characterized by a culture‐based approach, and fungal species were identified through rDNA‐ITS sequence analyses. From a total of 169 pure fungal isolates obtained from both the seed surface and internal tissues, we identified 16 distinct species (belonging to 10 genera) associated with maize seeds, all but one of which were ascomycetes. Among these species, seven were exclusively isolated from internal tissues, two species were isolated only from the seed surface, and another six species were isolated from both the surface and internal tissues. *Aspergillus niger* was consistently found under all storage conditions and dominated fungal communities with a relative abundance of 36%–100%. Species of *Fusarium* (9%–40%) and *Penicillium* (9%–20%) were also frequently isolated, but other species appeared sporadically and were isolated from fewer than three seed stocks. According to our results, while the overall incidence of fungal infection generally declined with storage time, there was no consistent association between seed storage time and fungal species richness or relative abundance; furthermore, the composition of the mycobiota associated with maize seeds was highly variable among the samples. The detection of the four major mycotoxigenic fungal genera, specifically *Aspergillus*,* Fusarium*,* Penicillium*, and *Alternaria*, was alarming, and the isolation of a potential controlling agent as well as information about their temporal occurrence will contribute to the management of mycotoxins in the future.

## 1 INTRODUCTION

A vast diversity of microorganisms colonize all organs of plants; these microorganisms constitute the plant microbiome and play an integral role in plant growth and health (Berg, Rybakova, Grube, & Koberl, 2016; Mendes et al., 2011; Rybakova et al., 2017; Sánchez‐Cañizares, Jorrín, Poole, & Tkacz, 2017). Recently, great efforts have been made to understand the plant microbiome, and the microbiota diversity of different tissues and developmental stages has been described for many plants, including major crops such as rice (Edwards et al., 2015), maize (Peiffer et al., 2013), barley (Bulgarelli et al., 2015), and soybean (Han et al., 2017; Liu et al., 2017; Sugiyama, Ueda, Takase, & Yazaki, 2014). Plant microbiota are hyper‐diverse and highly dynamic throughout the life history of plants (Peiffer et al., 2013; Sánchez‐Cañizares et al., 2017). However, the overwhelming majority of studies have focused on bacterial communities and on the microbiota associated with roots (rhizosphere) and above‐ground plant parts (phyllosphere). Much less attention has been paid to fungal communities (mycobiota); in particular, the mycobiota of seeds, one of the most important organs and a distinct developmental stage, remain poorly understood (Coleman‐Derr et al., 2016).

Seeds comprise micro‐scale ecosystems that sustain fungal communities and are highly ubiquitous and abundant. Many cultivated crops and wild plants have distinct fungal communities associated with seeds both externally and internally (Clay, 1990; Dhingra, Maia, Lustosa, & Mesquita, 2002; Ganley & Newcombe, 2006), showing mutualistic or pathogenic activities (Baker & Smith, 1966; Rodriguez, White, Arnold, & Redman, 2009; Stone, Bacon, & White, 2000). Pathogenic fungi are particularly problematic, as they are responsible for the re‐emergence of past diseases, the movement of diseases across administrative borders, and the introduction of diseases into new areas, posing tremendous threats to the global economy, food safety, and ecosystem health (Dean et al., 2012; Jones et al., 2008). In addition, some fungi that infect crop kernels also produce toxic secondary metabolites (mycotoxins), directly threatening human and livestock life (Bacon, Glenn, & Yates, 2008; Munkvold, 2003). Understanding the fungal communities associated with seeds is clearly of paramount importance in terms of disease control and food safety. However, our knowledge of the seed mycobiota is very limited.

Several factors may contribute to such a knowledge gap. First, many fungal species are associated with plant seeds during particular developmental stages. Based on their highly incomplete life histories, it is extremely difficult to identify fungal species because the most reliable diagnostic characteristics are lacking. In addition, many fungal species cannot develop sexual forms (teleomorphs) and fruiting bodies, which are key to species recognition, on culture media. Moreover, the seed mycobiota are not static but rather dynamic in both space and time (Baker & Smith, 1966; Han et al., 2017). Thus, developing a full understanding of the seed mycobiota is a challenging task that requires not only a reliable fungal recognition method but also time‐series samples of seed materials that capture the inter‐annual variability in fungal communities.

In this study, we examined the seed mycobiota of maize (*Zea mays*), one of the most globally important staple crop plants. To facilitate fungal species identification, we analyzed DNA sequences of the nuclear ribosomal internal transcribed spacer (rDNA‐ITS) regions of pure strains isolated from both seed surfaces and internal tissues. To reveal the inter‐annual variability in fungal communities, we examined seed samples with varying storage times ranging from 6 months to 12 years. Based on our DNA sequence data from the time‐series samples, we provide a comprehensive portrait of the seed mycobiota of maize, which may contribute to the future management of fungal diseases and mycotoxins in maize.

## 2 MATERIALS AND METHODS

### 2.1 Fungal sampling

A total of 11 time‐series seed stocks of the same maize cultivar, Zheng58, were cultivated and collected at the Zhangye Maize Stock Production Base (39.7°N, 100.2°E, 1437 m a.s.l.), Gansu Province, China, from 2004 to 2016. The region has a cold desert climate with a mean annual temperature of 7.3°C and an annual rainfall of 130 mm. Naturally, matured seeds were collected every year except for 2005 and 2015. Thus, the time of storage, or storage age, ranged from 6 months to 12 years at the time fungal isolation was performed (April 2016). New harvested seeds were bagged and stored in a sanitized warehouse at room temperature at the Zhangye Maize Stock Production Base.

To discriminate external (air‐borne) fungi and internal (seed‐borne) fungi, separate sampling procedures were employed. A total of 100 intact seeds from each of the 11 time‐series seed stocks were randomly selected for internal fungi isolation; 10 g (32–33 seeds) of each stock was used to first collect external fungi. Seed surface fungi were isolated using the suspension plating method (Mueller, Bills, & Foster, 2004). Briefly, 10 g of intact seeds were added to 90 ml of sterile distilled water to produce 10^−1^ (w/v) diluents. The seed slurry was shaken for 15 min and diluted to final concentrations of 10^−2^ and 10^−3^. Diluents were incubated in Petri plates (0.1 ml/plate) containing potato dextrose agar (PDA) medium with 40 μg/ml streptomycin and then incubated in the dark at 25°C for 4–7 days. All emerging colonies were subcultured to obtain pure cultures. To sample internal fungi, all 100 randomly selected intact seeds, including those that were used for external fungi collection, were treated with 1% sodium hypochlorite solution for 10 min followed by thorough rinsing with sterile distilled water three times. Dried seeds were incubated in Petri plates (10 seeds/plate × 10 plates/sample × 11 samples) containing PDA with 40 μg/ml streptomycin and were then incubated in the dark at 25°C for 14–28 days. Fungal isolates were repeatedly subcultured to obtain pure cultures. Pure fungal cultures were stored on agar slants of PDA at 4°C. The fungal strains were visually examined using a stereomicroscope and grouped based on morphological appearance and culture characteristics. All the isolates were deposited at the College of Plant Protection, Gansu Agricultural University, and at the College of Agriculture and Biotechnology, Hexi University.

### 2.2 DNA extraction, amplification, and sequencing

Fungal isolates of each seed sample with discernible differences in culture characteristics and morphological traits were subjected to molecular analysis. Fungal genomic DNA was extracted from mycelia using a Fungus Genomic DNA SK8259 Ezup Extraction Kit (Sangon Biotech Co., Ltd, Shanghai, China) following the manufacturer's instructions. To amplify rDNA ITS1‐5.8S‐ITS2 sequences, the primer pair ITS1/ITS4 (White, Bruns, Lee, & Taylor, 1990), which amplifies a ca. 600‐bp fragment, was used. The following PCR thermal profile was applied: an initial denaturation at 94°C for 4 min followed by 38 cycles of 30 s at 94°C, 30 s at 55°C and 30 s at 72°C, with a final extension at 72°C for 5 min. All the reactions were carried out in 25‐μL volumes composed of 1× reaction buffer, 1.5 mM MgCl_2_, 2.5 mM of each dNTP, 5 pM each primer, 0.6 units of Taq DNA polymerase (Promega Shanghai, China) and 30–50 ng of genomic DNA. The products were electrophoresed in 1% agarose gels in TAE buffer to check fragment lengths and detect the presence of nonspecific products. PCR products were purified with a QIAquickH PCR Purification Kit (QIAGEN Inc., Valencia, California) and were directly sequenced with ABI BigDye^™^ Terminator RR Mix (Applied Biosystems, Foster City, California) on an ABI 3130XL automated sequencer.

### 2.3 Phylogenetic analyses

Sequences were aligned using Clustal X 1.83 (Thompson, Gibson, Plewniak, Jeanmougin, & Higgins, 1997) and further inspected manually. Unique haplotypes were determined with DnaSP5.10 (Librado & Rozas, 2009) with alignment gaps taken into consideration. Homologous sequences in GenBank were queried using BLASTN 2.7.0 (Zhang, Schwartz, Wagner, & Miller, 2000) with the unique haplotypes. We downloaded homologous sequences with 100% coverage and ≥98% similarity in Blast searches to reduce the likelihood of incorrect species assignment. The homologous sequences were aligned with the unique haplotypes and subjected to phylogenetic analyses (Table S1). Phylogenetic analyses were performed with maximum likelihood (ML) and Bayesian approaches. ML analysis was carried out with the software PhyML 3.0 (Guindon et al., 2010). The best‐fit model of nucleotide substitution was identified in Modeltest 3.7 (Posada & Crandall, 1998) with the Akaike information criterion (AIC). Nodal support was assessed through 1000 bootstrap replicates. Bayesian analysis was carried out with MrBayes 3.2.6 (Ronquist et al., 2012) with the same model estimated from Modeltest. Analyses were initiated with random starting trees, and each analysis was run for 5 × 10^6^ generations with four Markov chains. Trees were sampled every 500 generations, and the first 25% of trees were discarded as burn‐in.

### 2.4 Mycobiota analysis

Although rDNA‐ITS sequences have been proposed as the primary marker for fungal species DNA barcoding (Schoch et al., 2012), there is no single unified yet stringent threshold for inter‐ and intraspecific variability in rDNA‐ITS sequences (Wang, Zang, Yin, Kang, & Huang, 2014); in some cases, rDNA‐ITS sequences are unreliable for species identification (Harder et al., 2013). We regarded isolates as belonging to the same species if they (1) had ≥98% sequence similarity, (2) formed the most derived monophyletic clades in a phylogenetic analysis, and (3) were highly similar in morphology and culture characteristics. For each seed stock, we calculated the number of distinct species isolated from the seed surface and internal tissue separately. Venn diagrams were generated to illustrate fungal community composition. For internal fungal communities, we treated genera as taxonomic units and examined changes in their relative abundance with storage time using stacked bar plots. The incidences were plotted as a function of storage time to examine community dynamics. All plots were performed and generated in the R (https://www.r-project.org/).

## 3 RESULTS

### 3.1 Phylogenetic inferences and fungal species recognition

**Figure 1:**
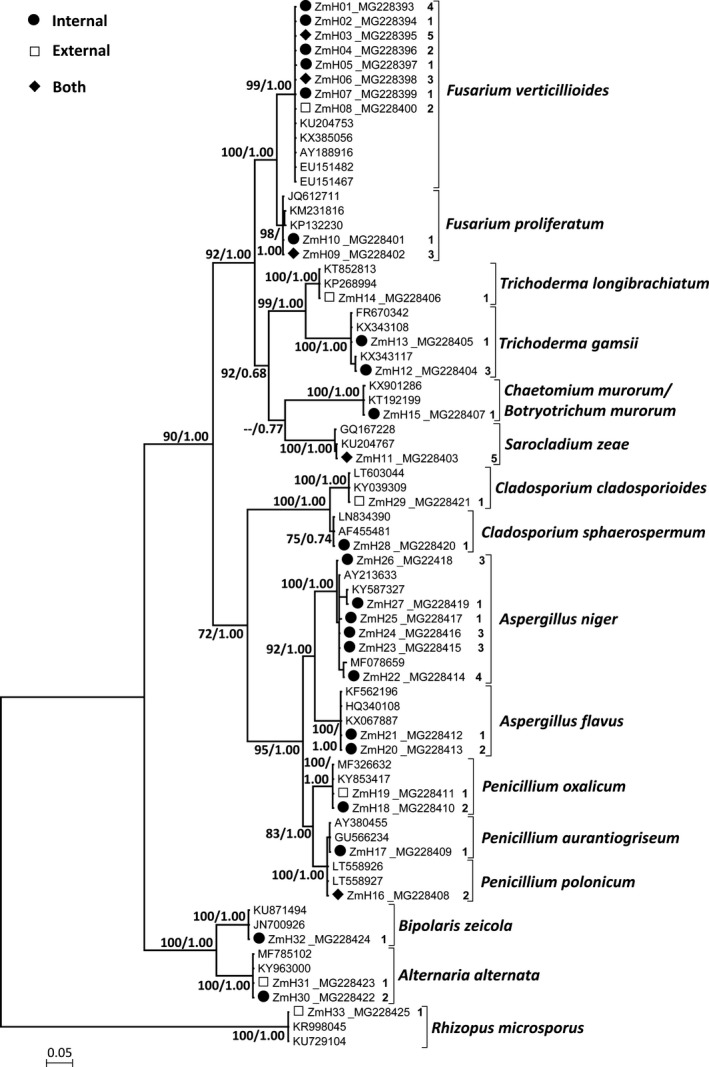
ML tree constructed using rDNA‐ITS sequences. Sequences for 65 pure‐cultured strains were collapsed into 33 unique haplotypes (ZmH01‐33). Black dots denote haplotypes for isolates from internal tissues, open squares denote haplotypes for isolates from the seed surface, and diamonds represent haplotypes that include isolates from both internal tissues and from the seed surface. The numbers following the haplotype names represent the number of strains sharing the haplotype. Numbers at the nodes refer to ML bootstrap support values from 1000 replicates and Bayesian posterior probabilities

A total of 169 fungal strains were isolated from 11 samples of maize seeds stored for 6 months to 12 years. Of these strains, 61 were isolated from internal tissues and 108 were associated with the seed surface. Pure‐cultured strains that were representative of all morphological and cultural variants were analyzed for rDNA‐ITS sequences. In total, 65 strains were sequenced, of which 53 strains were derived from internal tissues, and 12 strains were derived from the seed surface. The sequenced DNA fragments ranged from 520 to 630 bp in length. Considering mutations as well as differences caused by alignment gaps, the 65 raw sequences were collapsed into 33 unique haplotypes (ZmH01‐33), with frequencies ranging from one to five (Figure 1). The unique haplotypes have been deposited in GenBank under the accession numbers MG228393‐MG228425 (Table S1). We downloaded 39 homologous sequences and aligned them together with the 33 haplotypes, and a matrix comprised of 72 sequences was built for phylogenetic analyses. According to the AIC, the TrN model (Tamura & Nei, 1993) was the best fit for the alignment. The ML tree had a log likelihood of −5929.96. The topology of the ML tree and the Bayesian consensus tree appeared largely congruent, except for one of the internal nodes at which the ML tree differed from the Bayesian consensus tree by one subtree pruning and regrafting (SPR) move (Figure 1).

The high similarity between our haplotypes and their homologous sequences from GenBank made the assignment of species rather straightforward. Each species formed a strongly supported reciprocally monophyletic clade except for the extremely closely related species *Penicillium polonicum* and *P. aurantiogriseum* (Figure 1). The ITS sequences of these two species are highly similar; only a C to T transition was observed in the alignment. However, deletion of a T 35 bp upstream of the former point mutation was found to be fixed in *P. polonicum*. The genetic distances (*p*‐distances) between species ranged from 0.24% to 36.84% among fungi in Ascomycota, while the *p*‐distances ranged from 46.86% to 51.38% between species of Ascomycota and Zygomycota. The morphology and culture characteristics for all the assigned species were in accordance with their respective taxonomic descriptions.

### 3.2 Species diversity of the seed mycobiota

**Figure 2:**
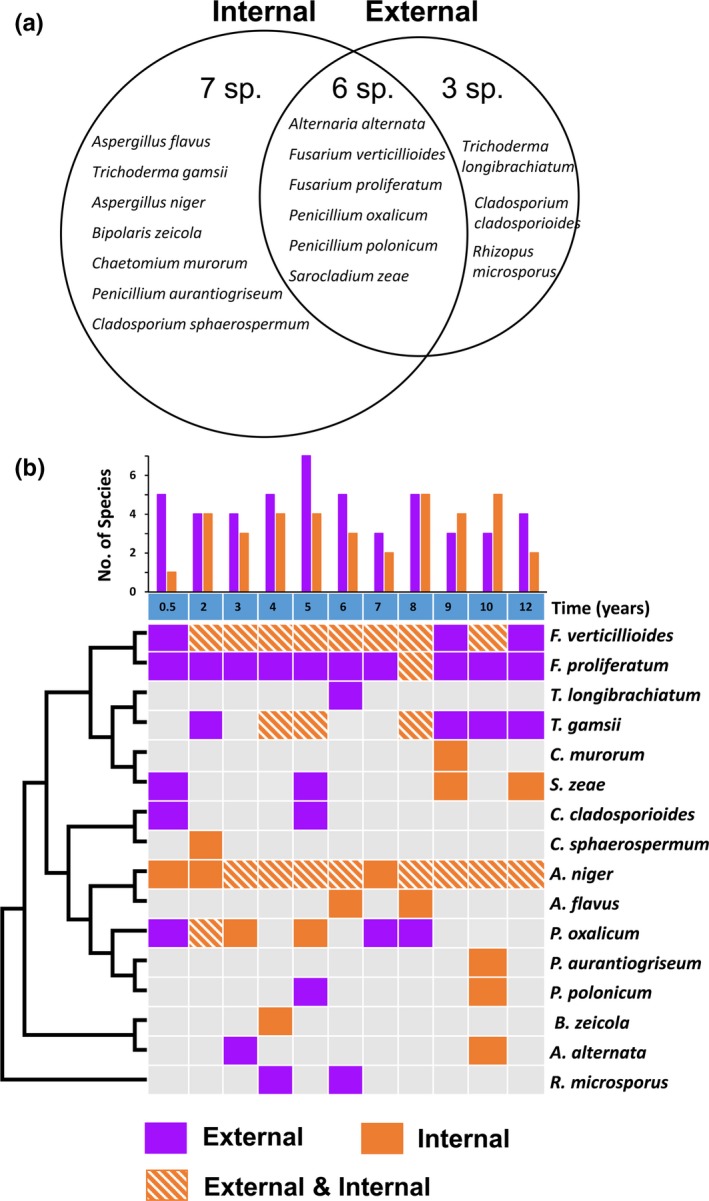
Fungal species associated with maize seeds. (a) Venn diagram showing distinct fungal species isolated from the internal, external and both parts of maize seeds. (b) Occurrence of fungal species in seed stocks with varying storage times (6 months to 12 years)

Based on the phylogenetic analysis results and morphological observations, we found a total of 16 distinct fungal species (belonging to 10 genera) associated with maize seeds. Among these species, 15 belonged to Ascomycota, and one species belonged to Zygomycota. Isolates of zygomycete species (*Rhizopus microsporus*) were only associated with seed surfaces. Among the ascomycetes, seven species (*Aspergillus niger*,* A. flavus*,* Bipolaris zeicola, Chaetomium murorum*,* Cladosporium sphaerospermum*,* P. aurantiogriseum* and *Trichoderma gamsii*) were exclusively isolated from internal tissue, two species (*Cladosporium cladosporioides* and *Trichoderma longibrachiatum*) were isolated only from the seed surface, and another six species (*Alternaria alternata*,* Fusarium verticillioides*,* F. proliferatum*,* Penicillium oxalicum*,* P. polonicum*, and *Sarocladium zeae*) were isolated both from the external surface and from internal tissues (Figure 2a).

Mycoflora compositions from each seed sample are plotted in Figure 2b. The number of species isolated from each sample ranged from three to seven for external fungal communities and from one to five for internal fungal communities. One species of *Aspergillus* (*A. niger*) and two species of *Fusarium* (*F. verticillioides* and *F. proliferatum*) were consistently found either externally or internally throughout the storage period, whereas other species were isolated sporadically. For most of the storage periods, *F. verticillioides* isolates were found both on external surfaces and in internal tissues, while *F. proliferatum* isolates were all associated with seed surfaces except in one 8‐year sample, in which it was isolated both from internal tissues and from the seed surface. Neither of the two *Fusarium* species were isolated from internal tissues of the newly harvested samples (storage age of 6 months) or in the oldest samples (storage age of 12 years), but both species were present on the surfaces of these seed samples. In contrast, *A. niger* isolates were always present within seeds of all ages, and *A. niger* was also isolated from the seed surface in 8 out of the 11 samples (Figure 2a).

**Figure 3:**
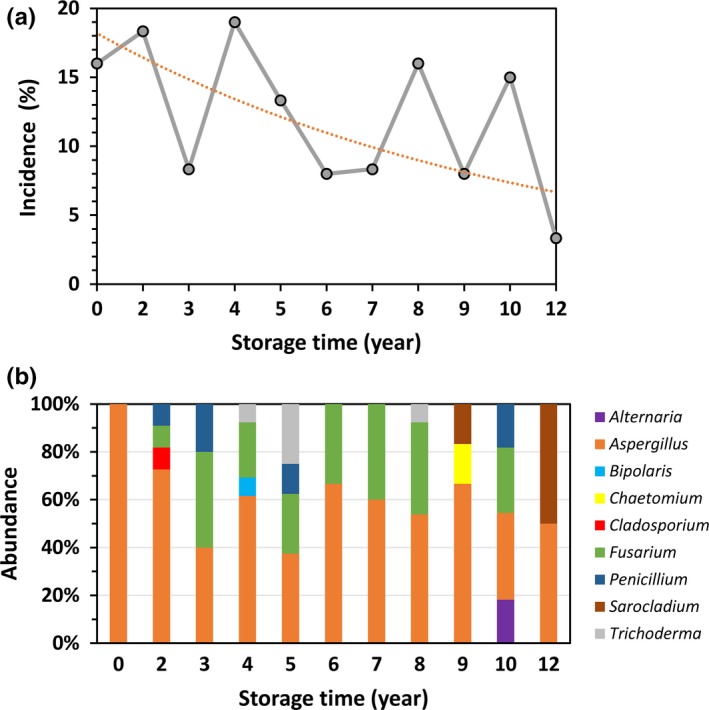
Incidence and composition of the mycobiota of maize seeds. (a) Proportions of seeds infected by fungi (incidences) for seed samples with various storage times (gray line) were fitted to an exponential distribution (dotted line). Although the incidence varied greatly with the storage time, a general trend in declining infection was clear. (b) Stacked bar plot of the composition and distribution of fungal taxa (collapsed to the genus level) from seed internal mycobiota

We further examined the incidence (proportion of seeds that were infected) and relative abundance of the internal fungi by storage time. The incidence of fungal infection varied with storage time (Figure 3a), ranging from 19% in the 4‐year samples to 3% in the 12‐year samples. However, a general trend of descending incidence was discernible (*y *=* *18.13*e* ^−0.08*x*^, *R²* = 0.34). A total of 13 species belonging to 9 distinct genera infected maize seeds. Figure 3b shows the relative abundance of each fungal genus in each storage stock. Maximum diversity (4 genera) was observed for the seed stocks with storage ages of 2, 5, and 10 years, while only a single genus (and a single species) was found in the newly harvested stock (0.5 years). *Aspergillus* clearly dominated (relative abundance: 36%–100%) the seed fungal community at all times, followed by *Fusarium* (9%–40%) at most postharvest time points with the exception of the 6‐month and 9‐year samples. *Penicillium* species were present in four stocks (2‐, 3‐, 5‐ and 10‐year samples) and comprised 9%–20% of the fungal communities. *Trichoderma* species occurred in the 4‐year (8%), 5‐year (25%), and 8‐year (8%) seed stocks, and *Sarocladium* occurred in the 9‐year (16%) and 12‐year (50%) stocks. The remaining four genera (*Alternaria*,* Bipolaris*,* Chaetomium*, and *Cladosporium*) appeared sporadically, occurring only in single stocks with relative abundances of less than 20%.

## 4 DISCUSSION

### 4.1 Low seed mycobiota diversity due to genetic purity and environmental uniformity

Molecular analyses of rDNA‐ITS sequences revealed that ascomycetes (15 species in 9 genera; 94%) dominated the mycobiota of the seed samples under study. Of these, only two species were restricted to seed surfaces, while the other 13 species colonized the internal tissues (Figure 2). Although we more extensively sampled seed stocks across multiple time points (100 seeds × 11 storage ages), the internal fungal species diversity did not appear higher than that reported in other studies. For example, Lević, Stanković, Krnjaja, Bočarov‐Stančić, and Ivanović (2012) found 15 seed‐borne fungal species in maize using a culture‐based approach similar to the approach we employed here; two species, *Acremonium strictum* and *Epicoccum* spp., were not isolated from our seed stocks. Lević et al. identified fungal species based only on morphological features, which might be less sensitive for recognizing species than the rDNA‐ITS sequences analyzed here, and thus likely underestimated species diversity. The species diversity of our samples was much lower when only a single time point (samples from a single year) was considered; a maximum of five species were found among our studied samples (Figure 2b).

Many factors may contribute to the low diversity of the mycobiota found in this study. Fungal diversity is determined by a combination of host‐specific factors and environmental factors. Among the host‐specific factors, the genetic background of seeds may be the most important. Species‐specific, cultivar‐specific, and even genotype‐specific components have been found within the plant microbiome (Andreote & Pereira E Silva, 2017; Berg & Smalla, 2009; Cardinale, Grube, Erlacher, Quehenberger, & Berg, 2015; Lundberg et al., 2012; Rybakova et al., 2017; Smalla et al., 2001), suggesting that the genetic background of plants plays a key role in modulating the composition and structure of the seed mycobiota (Oren, Ezrati, Cohen, & Sharon, 2003; Peiffer et al., 2013). Our multi‐year seed stocks were all the same maize variety (Zheng58) and thus shared the same host genetic background. Our observation of low fungal diversity is therefore consistent with the genetically homogeneous host plant we examined. Environmental factors also contribute to fungal assemblies and diversity, as they not only directly set the baseline conditions (i.e., ecological niche) under which a certain fungal species can survive but also indirectly affect the mycobiota by modulating the interactions between fungi and plants, between fungi and other microbes, and among different fungal species. The major environmental factors include temperature, humidity, and terroir, which are considered the most relevant to the seed mycobiota. Given our highly localized study area (all seed samples were produced within a field of 15 ha), the climatic and soil conditions are expected to be highly homogeneous and lack diversity. Such a small and environmentally uniform site would naturally foster a simple fungal community and microbiome in general. Our observation of uniformly low fungal diversity across all sampling years is completely in agreement with such an expectation. Based on all these points, we suggest that the low diversities of the mycobiota found here correspond to localized and crop‐specific or even cultivar‐specific factors.

### 4.2 Time‐series samples reveal the inter‐annual variation in dominant species

Another difference between the mycobiota revealed in this study and those from other studies pertains to the inter‐annual variation in dominant species. We found that species of *Aspergillus* (*A. niger*) overwhelming dominated the mycobiota in every sampled seed stock (Figure 3b). In terms of both incidence and relative abundance, *Aspergillus* surpassed *Fusarium* (*F. verticillioides* and *F. proliferatum*), the most prevalent fungal group associated with maize (Leslie, 1996; Lević et al., 2012; Nelson, 1992; Oren et al., 2003; Pamphile & Azevedo, 2002). The high abundance found here is in accordance with the fact that *A. niger* is one of the most common species of the genus *Aspergillus* and is ubiquitous in soil and indoor environments (Schuster, Dunn‐Coleman, Frisvad, & van Dijck, 2002). This species was the only fungal species we isolated from the internal tissues of newly harvested maize seeds, although five more species were collected from the seed surface (Figure 2b). Notably, we observed the highest incidence (16%) in the newly harvested seed stock and the lowest incidence (2%) in the oldest seed stock for this species.

*Fusarium* was the second most prevalent fungal taxon in our maize seed sample (Figure 2b). Specifically, we observed high incidences and relative abundances of *Fusarium* species during storage ages of 2 years to 10 years (Figure 3b). This finding suggests that conidia could survive at least 10 years in maize seeds. Such a long survival time might due to the fact that the seed stocks were kept in a seed bank and thus maintained an amicable condition for *Fusarium* fungi. In fact, we observed that the majority (ca. 80%) of seeds germinated successfully during the fungal isolation process. Of the two species identified here, *F. verticillioides* was consistently found both on the seed surface and within the internal tissues (Figure 2a). This species is the most frequently isolated species from maize seeds in other regions of world, for example, Argentina (Pacin et al., 2002), Mexico (Leyva‐Madrigal et al., 2015), and Serbia (Lević et al., 2012), and systematically infects all parts of plants (Oren et al., 2003). Isolates of *F. verticillioides* have also been demonstrated to be highly aggressive and capable of co‐infecting with other species of *Fusarium* (Leyva‐Madrigal et al., 2015). In contrast, another species, *F. proliferatum*, was primarily isolated from the seed surface; with only one exception, *F. proliferatum* infected seeds together with *F. verticillioides* in the 8‐year samples. Notably, neither *Fusarium* species infected newly harvested seeds, although both species were collected from the seed surface (Figure 2b). This phenomenon implied that infection might have occurred during storage.

Two additional mycotoxigenic fungal genera, *Penicillium* and *Alternaria*, were found in our seed stocks (Figures 1, 2, 3). *Penicillium* was the most species‐rich genus in our samples, with three isolated species. Among them, *P. oxalicum* appeared most frequently, while another two species, *P. aurantiogriseum* and *P. polonicum*, were isolated only from the 8‐year seed samples. A single *Alternaria* species (*A. alternata*) was also present only in the 8‐year seed stocks.

Fungal mycotoxins contaminate foods and animal feeds and have significant agricultural, epidemiological, and economic impacts, representing a worldwide problem (Chulze, 2010; Kabak, Dobson, & Var, 2006; Palumbo, O'Keeffe, & Abbas, 2008). The occurrence of the four major mycotoxigenic genera in our samples sends an alarming message that requires serious consideration of future seed handling practices. Notably, we isolated a species of *Chaetomium* (*C. murorum*) but no species of *Fusarium* in the 9‐year seed samples (Figures 2b and 3b). *Chaetomium* spp. have been reported to be seed endophytes that provide protection against *Fusarium* blight in oat seedlings (Soytong, Kanokmedhakul, Kukongviriyapa, & Isobe, 2001). The microbial interaction between species of *Fusarium* and *Chaetomium* within maize seeds represents an interesting issue worthy of further investigation.

Interactions between fungi and seeds are widespread in natural and managed ecosystems (Crist & Friese, 1993). While pathogenic fungi play contrasting roles in the structure, dynamics, and evolution of plant communities (Gilbert, 2002), plants also impose pivotal impacts on the mycobiota that reside within them. Seeds provide their mycobiota with microhabitats that are highly temporally variable. Consequently, it is reasonable to expect that fungal communities associated with seeds are also dynamic over time. The patterns of temporal change in fungal community composition in seeds reflect the inocula that existed at harvest as well as successive balancing among the population processes of immigration, emigration between internal and internal communities, and death for individual species; these processes are highly dependent on the physiological conditions of seeds, which are highly dynamic over time. In accordance with such expectations, we found sharp differences in species composition both on the surface and within seeds among maize seed stocks of different storage ages (Figure 2). In addition, the proportion of seeds infected by fungi tended to decline with seed storage age (Figure 3a). It is well known that the nutrient contents of maize decay with storage time. Moreover, the relative abundance of each fungal genus also changed with storage time. This point was clearly manifested by *Fusarium* species (Figure 3b). The dominant species and frequencies of particular fungal species on maize seeds have been shown to change over time (Lević et al., 2012), and microbiota variations have been found at different developmental stages in the seeds of other plants (Barret et al., 2015; Kovačec, Likar, & Regvar, 2016; Shade, Jacques, & Barret, 2017).

### 4.3 Perspective and implications

Infectious diseases remain one of the major threats to maize production, with fungi causing the majority of the reported maize diseases that affect roots, stalks, and kernels (Morales‐Rodriguez, Yanez‐Morales, Silva‐Rojas, Garcia‐de‐los‐Santos, & Guzman‐de‐Pena, 2007), not only reducing crop yield but also contaminating human food and animal feed. Mycobiota associated with seeds not only affect seed quality but also act as primary sources of the initial inocula for many fungal diseases. Determining the composition and relative quantities of species in seed mycobiota is clearly not only pivotal for understanding the occurrence and epidemiology of plant disease but also for providing opportunities for disease management using modern seed technologies, such as minimizing seed infection during field production and eradicating seed‐borne pathogens during postharvest processing (McGee, 1995). In the present study, we examined the species diversity and community composition of fungal assemblies both adhering to seed surfaces and colonizing the internal tissues of maize with storage times ranging from 6 months to 12 years. The inter‐annual variability in fungal communities revealed here represents a more comprehensive portrait of the maize seed mycobiota. These results from genetically uniform and environmentally homogenous samples are benchmark data describing the seed mycobiota of maize, to which future discoveries can be compared and for which likely contributing factors can be deciphered. The observed inter‐annual variation in the seed mycobiota implies that we should develop time‐aware strategies to effectively manage pathogenic and toxic fungi in practice.

We acknowledge that the cultivation‐dependent approach applied here, and in many other studies, is strongly biased to certain fungal groups, particularly ascomycetes, and might have only revealed a minor fraction of the seed microbiome fungal community. However, the fungal isolates obtained using such an approach allow for further experimentation to answer various questions concerning the biology, ecology and evolution contributing to our understanding of the underlying mechanisms that determine mycobiota composition and dynamics. These isolates also represent valuable materials for pathological and plant‐fungi interaction studies that will contribute to future disease management. In addition, the discovery of major pathogenic and mycotoxigenic fungal taxa as well as antagonistic species in this study signifies that the cultivation‐dependent approach remains an effective tool for studying the seed mycobiota because it provides invaluable insights that are impossible with cultivation‐free approaches. Future work should include the targeted examination of fungal species from large samples of seeds to understand the relationship between seed physiological characteristics and fungal survival rates, and the fungal communities of the same seed stocks for multiple time‐intervals should be re‐isolated and re‐characterized to reconstruct the mycobiota dynamics of maize seeds. Such studies may lead to novel strategies for the prevention and management of disease and mycotoxins in maize.

## CONFLICTS OF INTEREST

None declared.
