# Supplementary material for: Mycobiota of maize seeds revealed by rDNA‐ITS sequence analysis of samples with varying storage times
Source: Microbiologyopen. 2018 Mar 23;7(6):e00609. doi: 10.1002/mbo3.609 (PMC6291794; doi:10.1002/mbo3.609)
Supplement: Supplementary file 1 [file MBO3-7-e00609-s001.pdf]

Table S1. Haplotypes, GenBank accession numbers, and host of sequences used in phylogenetic analyses. Haplotypes ZmH01-33 were sequenced from 65 fungal isolates in this study.

Species	Isolates/Haplotypes	Accession	Sources
Fusarium verticillioides	ZmH01-08	MG228393-400	Maize seeds
	Bt3S2	KU204753	Maize
	DET-51	KX385056	Seeds of peroba rosa
	N.A.	AY188916	Animal and plant
	SA3	EU151482	unknown
	A1	EU151467	unknown
Fusarium proliferatum	ZmH09-10	MG228401-402	Maize seeds
	MB-013	JQ612711	Amaryllis
	CBS 189.38	KM231816	unknown
	M14	KP132230	Human sputum
Trichoderma	ZmH14	MG228406	Maize seeds
longibrachiatum	NFCF081	KT852813	*Lentinula edodes*
	OUCMBI110082	KP268994	Marine macroalgae
Trochoderma gamsii	ZmH12-13	MG228404-405	Maize seeds
	IUVV D5P4-11	FR670342	Grapes
	AV10	KX343108	Soil
	LIT3-9.1	KX343117	Soil
Chaetomium murorum	ZmH15	MG228407	Maize seeds
	B1/3/VF	KX901286	Books
	00153-1	KT192199	Arborvitaes
Sarocladium zeae	ZmH11	MG228403	Maize seeds
	NRRL:47823	GQ167228	Maize
	Bt4S3	KU204767	Maize
Cladosporium	ZmH29	MG228421	Maize seeds
cladosporioides	N.A.	LT603044	Library materials
	CCTU1049	KY039309	Soil
Cladosporium	ZmH28	MG228420	Maize seeds
sphaerospermum	wb311	AF455481	Nasal mucus
	UTHSC DI-13-237	LN834390	Clinical samples
Aspergillus niger	ZmH22-27	MG228414-419	Maize seeds
	UWFP 515	AY213633	unknown
	77	KY587327	*Sceletium tortuosum*
	MRC200804	MF078659	Lentil
Aspergillus flavus	ZmH20-21	MG228412-413	Maize seeds
	PW2953	KF562196	unknown
	MUM 10.220	HQ340108	Grapes
	Sichuan-Rfsb11	KX067887	Rice false smut ball

Penicillium oxalicum	ZmH18-19	MG228410-411	Maize seeds
	IHBF 2338	MF326632	High altitude lakes
	SPL16055	KY853417	Sweet potato
Penicillium aurantiogriseum	ZmH17	MG228409	Maize seeds
	FRR 971	AY380455	unknown
	D8	GU566234	Rhizosphere
Penicillium polonicum	ZmH16	MG228408	Maize seeds
	DI16-104	LT558926	Clinical samples
	DI16-105	LT558927	Clinical samples
Bipolaris zeicola	ZmH32	MG228424	Maize seeds
	TR572	KU871494	Maize fields
	H05-505-1	JN700926	Sorghum
Alternaria alternata	ZmH30-31	MG228422-423	Maize seeds
	Alt 05	MF785102	Harvest grapes
	SJSJ-3-2	KY963000	*Cephalotaxus oliveri*
Rhizopus microsporus	ZmH33	MG228425	Maize seeds
	ZJPH 1308	KR998045	Soil
	ATCC 22959	KU729104	unknown
